# Standards of practice for peripheral nerve blocks at a tertiary care center in a low-middle income country– a prospective observational study

**DOI:** 10.1186/s12871-025-03125-8

**Published:** 2025-05-24

**Authors:** Heena Purshotamdas Bhupta, Priyanka Kini, Nisha Sara M. Jacob

**Affiliations:** 1 Department of Anesthesia, Rainbow Childrens’ Hospital, Banjara Hills Road, Hyderabad, India; 2 Department of Anesthesia, Kasturba Medical College, Manipal, Manipal Academy of Higher Education, Manipal, Karnataka India

**Keywords:** Peripheral nerve block, Practice pattern, Anesthesia, Ultrasound

## Abstract

**Background:**

Peripheral nerve blocks (PNB) have gained tremendous interest as a means of providing anesthesia and analgesia. Heterogeneity exists in the standards of practice (SoP) for PNB, especially in resource limited settings. Therefore, this study aimed to determine the SoP for administration of PNB at a tertiary care, University teaching hospital, in a low-middle income country (LMIC).

**Methods:**

This was a prospective observational study conducted between September 2021 and March 2023. The SoP were collected through either direct or indirect observation, using a self-developed proforma. Data were collected for various domains and were mapped to structure, process and outcome. They were then benchmarked to identify best practices and deficiencies.

**Results:**

A total of 386 PNBs were recorded, of which 196 (50.8%) were directly observed. Majority were administered to low-risk individuals without comorbidities (61.9%). In the structure metrics resuscitation equipment was available in 76% while availability of resuscitation drugs (29%) was identified as needing improvement. Individual components of process metrics such as intravenous access, use of personal protective equipment, ultrasound guided block technique and use of short bevel needles exceeded the 95% benchmark, while components such as monitoring (54%), aseptic site preparation (76.5%), the Stop Before You Block procedure (61.9%) and use of block additives (70.2%) fell short of the benchmark. Postoperative multimodal analgesia prescription (4.7%) was identified as needing improvement. Of the outcome metrics, while immediate post-block complications (0%) and conversion rate to general anesthesia (8.3%) exceeded the benchmark (< 10%), cumulative toxic dose was exceeded (36.6%) when a mixture of local anesthetics was used.

**Conclusion:**

Standards of practice for structure, process and outcome were partly achieved for PNB at this tertiary care center in a low-middle income country. Strategies to improve SoP have been proposed and will need to be evaluated in future quality improvement initiatives.

**Supplementary Information:**

The online version contains supplementary material available at 10.1186/s12871-025-03125-8.

## Background

Nearly five billion people do not have access to safe and affordable surgical and anesthesia care. The Lancet Commission on Global Surgery believes that safe and affordable surgical and anesthetic care are vital to reducing premature death and disability [1]. These, however, are challenging in low-middle income countries (LMICs). With the goal to reduce both catastrophic and out-of-pocket costs from surgeries in LMICs, it is important that individuals have access to safe and appropriate anesthesia and analgesia. One of the needs for safe anesthesia care is *“Infrastructure*,* equipment and supplies necessary to perform safe general anesthesia*,* loco-regional anesthesia….”.* It is in this context that we explore the standard of practice (SoP) of peripheral nerve blocks (PNB).

PNB are widely accepted for surgical anesthesia and analgesia, with well-established clinical benefits, and have evolved from landmark to ultrasound (USG) guided techniques, with unique advantages and challenges [2]. They are a safe alternative in high-risk populations where general anesthesia may be avoided in the presence of compromised cardiorespiratory function or multi-organ failure [3–6]. With evidence suggesting that PNB reduces the costs incurred for analgesia in the perioperative period, the long-term implications of high quality PNB cannot be underestimated [7]. In particular, PNB in low-resource settings has definitive advantages of avoiding airway manipulation during general anesthesia (GA), enhancing cost-effectiveness in areas where resources and personnel are already scarce, and augmenting optimal postoperative analgesia, especially in areas where access to opioids is severely limited due to legal and regulatory restrictions [8, 9]. While there is no doubt that training, infrastructure, equipment and drugs are at a premium in low-resource settings, the importance of safety, quality and efficacy in these settings cannot be understated [10]. Therefore, it is vital for best practices on safety, quality, and efficacy for PNB to be advocated [2]. These are constantly evolving and may not be universally followed. Thus, there is a need to understand current practices of PNB administration to identify lacunae that exist and develop strategies to improve PNB administration so that individuals have access to safe anesthesia and analgesia in LMICs. Therefore, the objective of this study was to understand the SoP for PNB in a LMIC.

## Methodology

This was a prospective observational study, conducted at a tertiary care university teaching hospital on participants who received PNB between September 2021 and March 2023. Adult participants who underwent elective and emergency procedures with PNB were eligible to participate and were recruited through purposive sampling. All other forms of anesthesia without PNB, and participants involved in other studies were excluded. The study was commenced after Institutional Ethics Committee approval and trial registration. All participants provided written informed consent prior to enrolment. The STrengthening the Reporting of OBservational studies in Epidemiology (STROBE) guidelines were followed for the reporting of this study [11].

A self-developed proforma was given to consultant anesthetists immediately after the block completion to collect data regarding details of PNB administration. Additionally, the anesthetic charts were reviewed by the principal investigator for any missing data. The content of the proforma comprised of six domains that were mapped to the Donabedian model of structure, process and outcomes [12] (Additional File, Table A). The principal investigator aimed to directly observe as many procedures as feasible (direct observation) to ensure data quality maintenance and to avoid reporter and recall bias.

### Statistical analysis

An *a*-priori minimum sample size of 384 was determined using the estimation of proportions for a desired level of precision of 0.05, with at least 50% samples captured (*p* = 0.5 and q = 1-p), and 95% confidence interval (Z = 1.96).

Data were analyzed using JAMOVI (v. 2.5.7). Descriptive analysis was used to represent the demographic data. Quantitative parameters were represented as mean *+* standard deviation (SD). Categorical and nominal data were expressed as frequencies and percentages. Adherence to each performance measure was benchmarked and distance from the estimated value was calculated. General linear models were used to determine the association between demographic characteristics with outcome metrics. Statistical significance was considered when *p* < 0.05.

Based on consensus amongst the investigating team, standards were said to be met when performance and quality parameters were adhered to in all observations (Table 1). Based on the percentage cut-offs, performance of the measures was categorized as excellent (green; meeting/exceeding the benchmark value, as described in Table 1), good (yellow; not meeting the benchmark value by 15%), average (pink; not meeting the benchmark value by 15–45%) and needs improvement (red; not meeting the benchmark value by > 45%).

**Table 1:** Benchmarking quality and performance measures

Parameter	Benchmark
**Structure metrics**	
Availability of resuscitation equipment (Anesthesia machine checked and in working order, oxygen source, oxygen tubing, manual resuscitator / self-inflating bag, face mask, intubation tray with working laryngoscopes)	Average of components available in > 95% of PNB procedures
Availability of resuscitation drugs (Atropine, mephentermine/ephedrine, glycopyrrolate adrenaline, midazolam, intralipid)	Average of components available in > 95% of PNB procedures
**Process metrics**	
Monitors attached (Non Invasive Blood Pressure (NIBP)/pulse oximeter/ Electrocardiogram (ECG ) leads)	Average of components used in > 95% of PNB procedures
Venous access	Accessed in > 95%
Aseptic procedures– Use of PPE (Cap, mask, sterile gloves, sterile gown)	Average of components used in > 95% of PNB procedures
Aseptic procedures– Site preparation (Skin preparation, sterile drapes, sterile cover for ultrasound, sterile lubricant gel)	Average of components used in > 95% of PNB procedures
Follows “Stop Before You Block”	Followed in > 95% of PNB procedures
Use of ultrasound	Used in > 95% of PNB procedures
Use of short bevel needles block needles	Used in > 95% of PNB procedures
Use of additives in block	Used in > 95% of PNB procedures
Prescription of postoperative analgesia	Performed in > 95% of PNB procedures
Multimodal analgesia prescription for postoperative analgesia (prescription of *>* 2 analgesics)	Prescribed in > 95% of PNB
**Outcome metrics**	
Conversion to GA in PNB planned under block alone	Occurs in < 10% of PNB planned under block alone
Presence of immediate post-block complications (related to local anesthetic systemic toxicity or specific to block site)	Occurs in < 10% of PNB procedures
Exceeding cumulative dose of local anesthetic (LA) toxic limit (i.e., when more than one LA is used, the dose is additive, and the sum must be within toxic limit for the weight of the patient)	Occurs in < 10% of PNB procedures

Abbreviations: PNB- peripheral nerve block, GA– General anesthesia; PPE– Personal protective equipment

## Results

A total of 386 participants were screened for eligibility and recruited (men: 264; 68.4% and women: 122; 31.6%). Majority (239, 61.9%) were low risk without comorbidities (Table 2). No statistical significance was observed between body mass indices for men and women (24.9 ± 3.63 Kg/m^2^ and 24.6 ± 3.88 Kg/m^2^; *p* = 0.539). Of the 386 blocks, 196 (50.8%) were directly observed.

Locations for PNB administration were in the preoperative holding bay (PHB) (*n* = 338, 87.5%), within the operating room (*n* = 32, 8.2%) and alternate locations (*n* = 16, 4.1%); χ^2^ = 512, *p* < 0.001. There was no correlation observed between the nature of surgery (i.e., emergency versus elective) and the location of PNB administration (Spearman’s rho = 0.09; *p* = 0.051). The most common anatomical site of PNB was the upper limb (289; 74.8%) followed by the lower limb (79; 20.4%). Other anatomical sites are described in Additional File, Table B.

**Table 2:** Participant characteristics and preoperative assessment

Variable	Value
Age in years, mean ± SD	46.3 ±16.1
Weight in Kg, mean ±SD	66.7 ±12
Height in meters, mean ±SD	1.64 ±0.08
BMI in Kg/m^2^, mean ± SD	24.8 ±3.7
ASA PS, n (%)	
I II III IV	239 (61.9) 85 (22) 55 (14.2) 7 (1.8)
Nature of surgery, n (%)	
Elective Emergency	204 (52.8) 182 (47.1)

Abbreviations: ASA PS- American Society of Anesthesiologists Physical Status, BMI- Body Mass Index

Pre-operatively, 22 participants (5.6%) were on antithrombotics, the majority of whom underwent emergent procedures (*n* = 15/22, 68.2%). In 13/22 (59.1%), the antithrombotics were continued. Procedural sedation was used in 31/386 (8%) participants. Among them, the most used drugs were fentanyl (*n* = 24, 77.4%), midazolam (*n* = 5, 16.1%), ketamine (*n* = 1, 3.2%) and propofol (*n* = 1, 3.2%). This was not affected by direct observation (Spearman’s rho = 0.004, *p* = 0.93).

### Structure metrics

**Fig. 1:**
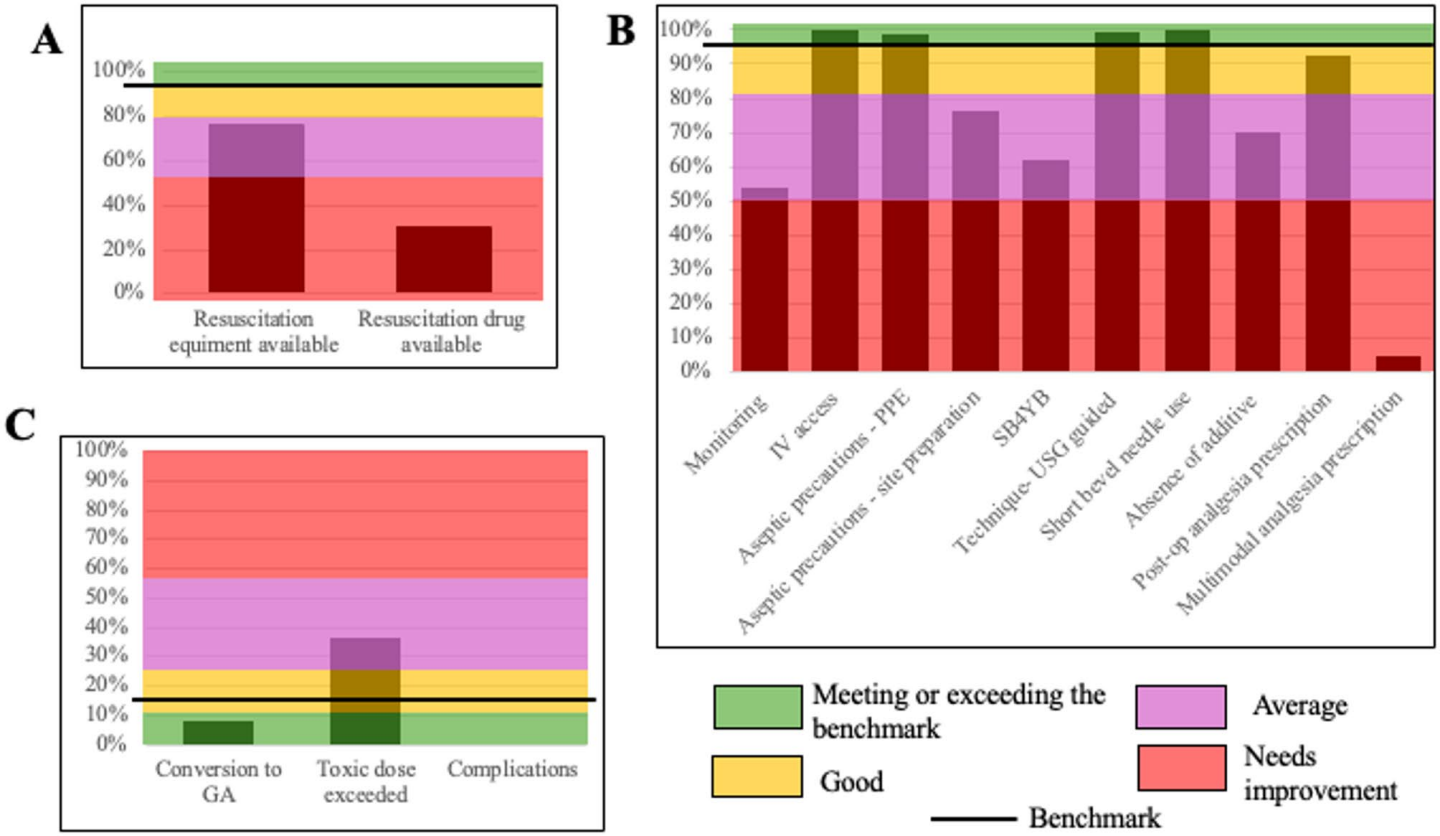
Benchmarking of Structure (**A**), Process (**B**) and Outcome (**C**) metrics Abbreviations: IV- intravenous; PPE– personal protective equipment; SB4YB– Stop Before You Block; USG– ultrasound; GA- general anesthesia; Post-op– postoperative Legend: Panels A, B and C depict the achieved standards of practice for PNB. The solid line represents the a priori benchmarks as described in Table 1. The color coding used describes the degree to which these standards have been achieved: excellent (green; meeting/exceeding the benchmark value, as described in Table 1), good (yellow; not meeting the benchmark value by 15%), average (pink; not meeting the benchmark value by 15–45%) and needs improvement (red; not meeting the benchmark value by > 45%)

The overall availability of resuscitation equipment was average (76%) (Fig: 1A). Individual components such as availability of oxygen source and tubing, manual resuscitator and face mask exceeded the benchmark (Table 3), while others such as availability of an anesthesia machine in the vicinity and an intubation tray with working laryngoscopes were lacking. Availability of resuscitation drugs in the PNB vicinity required improvement (overall 29.6%) (Fig: 1A). None had intralipid in the vicinity of block administration (Table 3).

**Table 3:** Quality parameters related to Preparation for block and aseptic precautions

**Availability of resuscitation equipment**, ***n*** (**%**)	
Oxygen tubing Oxygen source Manual resuscitator / self-inflating bag Face mask Anesthesia machine checked and in working order Intubation tray with working laryngoscopes	380 (98.4) 375 (97.2) 367 (95.1) 366 (94.8) 139 (36) 134 (34.7)
**Availability of resuscitation drugs**, ***n*** (**%**)	
Atropine Mephentermine/ ephedrine Glycopyrrolate Adrenaline Midazolam Intralipid	155 (40.1) 155 (40.1) 155 (40.1) 155 (40.1) 67 (17.4) 0 (0)
**Monitoring equipment and venous access**, ***n*** (**%**)	
SpO_2_ probe NIBP ECG leads Venous access “STOP BEFORE YOU BLOCK” performed	356 (92.2) 135 (35) 135 (34.7) 386 (100) 239 (61.9)
**Aseptic precautions - personal protective equipment**,** n (%)**	
Cap Mask Sterile gloves Sterile gown	386 (100) 386 (100 384 (99.5) 369 (95.6)
**Aseptic precautions - site preparation**,** n (%)**	
Skin disinfection performed Povidone iodine + spirit Chlorhexidine Surgical spirit Povidone iodine Sterile drape Sterile cover for USG Sterile lubricant gel	386 (100) 358 (92.7) 23 (5.9) 3 (0.7) 2 (0.5) 383 (99.2) 380 (98.4) 33 (8.5)

Abbreviations: SpO_2_- pulse oximeter; NIBP- non-invasive blood pressure; ECG-electrocardiogram; USG– Ultrasound

### Process metrics

All participants had venous access in situ before block administration. Pulse oximeter was the sole monitor in most cases (*n* = 356, 92.2%). Overall adherence to monitoring modalities (54%) fell short of the benchmark (Table 3), as did the “Stop Before You Block” (SB4YB) exercise (61.9%), the understanding of which varied in the directly observed blocks. Overall adherence to personal protective equipment (PPE) was higher than the benchmark, 98.7% (Fig:1B). Greater variation was seen in the aseptic procedure components related to site preparation (overall 76.5%), particularly with the use of sterile gel (Table 3; Fig: 1B).

USG was used in 383 cases (99.2%), which well exceeded the benchmark (Fig:1B). Landmark with USG (*n* = 2, 0.5%) or landmark alone (*n* = 1, 0.2%) was used in the remaining. All but two practitioners (intravenous canula (*n* = 1) and hypodermic needle (*n* = 1)) used short bevel needles. All used the in-plane needling technique. Majority of the participants received levobupivacaine with lignocaine-adrenaline (*n* = 281, 71.7%), followed by levobupivacaine (*n* = 52, 13.4%), bupivacaine with lignocaine-adrenaline (*n* = 25, 7.5%), ropivacaine (*n* = 15, 3.8%), bupivacaine (*n* = 8, 2.07%) and lignocaine-adrenaline (*n* = 5, 1.2%). No additives were used in most PNB (*n* = 271, 70.2%). When used, dexmedetomidine (*n* = 56, 49.1%), dexamethasone (*n* = 46, 40.3%), clonidine (*n* = 8, 7.01%), fentanyl (*n* = 3, 2.6%) and buprenorphine (*n* = 1, 0.89%) were preferred. The use of short bevel needles superseded the benchmark while use of additives in the blocks was average (Fig: 1B).

Postoperative analgesia was prescribed by the anesthesiologists in 356 (92.2%) participants. Paracetamol was prescribed in all these participants, with diclofenac, nalbuphine and tramadol additionally prescribed in eight (2.1%), six (1.6%) and four (1%) participants respectively. Multimodal analgesia prescription (i.e., prescription of ≥ 2 analgesics) was inadequate (4.7%, *n* = 17/356) (Fig: 1B).

### Outcome metrics

The plan of anesthesia was most often PNB (*n* = 263, 68.1%), followed by PNB + general anesthesia (GA) (*n* = 102, 26.4%) and PNB + neuraxial anesthesia (*n* = 21, 5.4%). In 13 (12.7%) procedures planned under PNB + GA, PNB was administered after induction. Of the 263 procedures planned under PNB, 22 (8.3%) were converted to GA, which was within the desired benchmark range (Fig: 1C). The commonest reason was ‘patchy block’ (*n* = 13, 59%); other reasons recorded were ‘effect diminished with time’ (*n* = 5, 22.7%), failed block (*n* = 2, 9.1%) and uncooperative patient (*n* = 2, 9.1%). Direct observation of the procedures did not impact the conversion of blocks to GA (χ^2^ = 0.994 *p* = 0.608). Intraoperative analgo-sedative supplementation not amounting to GA, was required in 17% (*n* = 41) of participants who underwent surgery solely under PNB.

A combination of local anesthetics (LA) was used in 306 (79.2%) participants. Among these, the total additive dose exceeded the toxic limit in 112/306 (36.6%). This was above the benchmarked level of 10% (Fig: 1C). 85/386 participants were unable to stand and therefore not weighed; estimation of whether the toxic dose was exceeded could not be performed in these participants. No clinical effects of local anesthetic systemic toxicity (LAST) were reported. We explored the role of gender, age, body mass index (BMI), American Society of Anesthesiologists (ASA) classification, use of mixture of LA, elective vs. emergent nature of surgery, and whether the blocks were directly observed or not, on the total dose exceeding the toxic limit and found BMI (OR = 1.15; 95% CI: 1.06–1.25; *p* < 0.001) and use of mixture of LA (OR = 8.77; 95% CI:2.99–25.73; *p* < 0.001) to significantly impact this outcome.

No immediate post-block complications as related to LAST or specific to the PNB administered were observed in any participant.

## Discussion

This survey identified SoP for PNB in a tertiary care teaching hospital in a LMIC. Heterogeneity in practice was observed in various domains, with individual metrics achieving benchmark standards to varying degrees.

Most participants on antithrombotics underwent emergent surgeries. The underlying co-morbid illness may have precluded discontinuation. Although antithrombotics were continued in 59.1%(*n* = 13) participants, all received superficial blocks. No immediate post-block complications were observed. While guidelines for neuraxial anesthesia in patients on antithrombotics are well established [13, 14], these are often considered restrictive for application to PNB. Recent guidelines suggest superficial PNB are unlikely to produce clinically significant bleeding; therefore, antithrombotic cessation may be unnecessary [14]. On the other hand, deep PNB at non-compressible sites require withdrawal of antithrombotics for appropriate durations [13] and PNB should be considered only after risk-benefit assessment, documentation, and informed consent.

The focus on PNB has shifted from merely performing the block to safe and efficacious execution. To this end, guidelines stipulate availability of appropriate drugs and equipment and protocols to manage any unanticipated situations [15]. Pre-block venous access is mandatory, and the SB4YB procedure is advocated to prevent wrong-sided blocks (WSB) [2, 15]. A mandatory pre-block checklist ensures availability of appropriate drugs and resuscitation equipment.

In our survey, lacunae were identified primarily with the availability of resuscitation drugs rather than resuscitation equipment (structure metrics). In particular, intralipid was unavailable in the vicinity of block administration, although best practices advocate it [2, 15]. The anesthetists may believe the low incidence of LAST may not warrant keeping intralipid in the vicinity. Despite this, preparedness to deal with LAST at this center was demonstrated by the presence of a flowchart attached to each USG machine with the protocol to be followed in the event of a LAST, along with a picture of where the intralipid was stored.

The process metrics fared reasonably well. Venous access was universally secured. Although pulse oximeter was used in most participants, majority did not have an electrocardiogram or blood pressure monitoring during block administration. Variable availability of monitoring equipment in the PHB (the commonest location for block administration at this center) may have contributed to these deficiencies. Scarcity of equipment outside the operating room, shared resources between locations and malfunctioning equipment are a reality in many low-resource settings [8–10]. Often, the cast-offs are relegated to non-operation room areas. It is important to distinguish between ‘unavailable’ and ‘un-used’ resources, which were not differentiated in this study. Since most participants belonged to ASA Physical Status I and received superficial blocks, we speculate that anesthetists may have considered PNB as ‘low risk’ procedures which did not warrant the use of all standard monitors (i.e., ‘un-used’ resources). Furthermore, the pressures of a fast-moving operating list would tempt one to cut corners. The widespread use of USG, as was evident in this survey, might give the anesthetist the impression of a copacetic state. Assessment of knowledge and attitude of anesthetists may shed light on these areas. Establishment and efficient functioning of a “block room” near the operating room with the necessary drugs and equipment offers advantages such as enhanced productivity, increased theatre efficiency, a safe and stress-free environment for PNB administration and opens avenues for educational and research opportunities [16].

No WSB, recognized as a ‘never event’, were reported in our survey perhaps reflecting its low incidence (1.28 per 10,000 blocks) [17]. The SB4YB initiative, following the ‘prep, stop, block’ steps, with clear site demarcation and checklists have been advocated in the routine pre-block preparation [18]. Although SB4YB was performed in 61.9% cases, there was heterogeneity among the directly observed blocks, which may reflect individual variation in understanding of this procedure. Educating, enabling, and empowering operating room assistants in the execution of SB4YB will strengthen this endeavor. Frequent training and updates, memory aids in the block area and institutional policies with quality improvement measures may be undertaken to prevent WSB [19–22].

Best practices recommend aseptic precautions be instituted for PNB administration [2]. Our overall compliance with aseptic precautions was high (average of 87.3%), with variations noted in individual components (Table 3; Fig: 1). Although no catheters were inserted, full personal asepsis (which is recommended for PNB with catheter insertion) was followed in majority of the cases. Skin disinfection was universally followed. However, the disinfecting agent varied (Table 3). This may not be a major concern since superiority of chlorhexidine over povidone iodine has not been demonstrated [23]. Importantly, since both are neurotoxic, lower concentrations and complete drying are recommended [24, 25]. Non-sterile gel was used in most cases, which constituted a breach in sterility. This may reflect non-availability of sterile gel or lack of awareness among anesthetists. Frequent updates reinforcing aseptic precautions and quality improvement initiatives with audits would improve existing standards [26].

Most PNB were performed with USG, suggesting this is the standard of care. Safety is enhanced when USG is combined with a peripheral nerve stimulator [27]. However, none of the practitioners combined both modalities, perhaps due to unfamiliarity with, or unavailability of a peripheral nerve stimulator. Most practitioners used short bevel needles and practiced in-plane needling, which are strategies that are recommended to prevent peripheral nerve injury (PNI). Another strategy recommended is monitoring of injection pressure to < 15 psi [28]. Since the injection pressure monitor was unavailable at our center, monitoring of injection pressure was not performed. In a low-resource setting, a practical and cost-effective alternative could be the Compressed Air Injection Technique (CAIT), where 50% compression of air during injection limits injection pressures to 20 psi, a level above which persistent neurological damage has been observed in animal models [29].

PNB after GA is often considered unsafe since the anesthetist depends on the patient being a ‘live monitor’ to detect paresthesia, thereby averting PNI. Early detection of symptoms of LAST is possible in an awake patient. With the skilled use of USG, PNB under GA is a reasonable option in select, low-risk patients [2, 30]. Under anesthesia, the patient is comfortable, particularly when multiple PNB are required, thereby enhancing PNB acceptance. In this study, for most procedures planned under GA + PNB, PNB was administered before GA. This may simply reflect efforts to save operating room time or may be a conscious effort to enhance safety of PNB. Intraoperative analgo-sedative supplementation was required in 17% of participants who underwent surgery solely under PNB. Possible reasons include inadequate time for optimal block effect, patient anxiety (which may highlight perception of sensations unrelated to pain), or breakthrough tourniquet pain.

Postoperative analgesia was not prescribed in 7.2% of participants by the anesthetists. At our center, analgesia may be prescribed by surgeons or anesthetists. This may shift the burden of prescription on to the surgeon rather than the anesthetist, which could impact the perceived need for postoperative analgesia prescription by the anesthetist. Furthermore, since either the surgeon’s or the anesthetist’s prescription may be followed, discrepancy may exist between the anesthetist’s prescription and the analgesia administered. This was, however, not analyzed in this study.

Among those prescribed analgesics, paracetamol was universally prescribed. PNB offers analgesia in the immediate postoperative period which may lull the anesthetist with a false assurance of adequate postoperative analgesia. With inadequate analgesia, rebound pain may complicate the postoperative period [31]. This can lead to paradoxically increased opioid consumption, delayed recovery and hospital discharge (which can place additional financial burden) and potentially induce chronic pain syndromes [32, 33]. Considering additives were not used in most cases (*n* = 272, 70.4%), paracetamol as the sole analgesic may have been insufficient to ensure adequate analgesia in the postoperative period. Strategies such as multimodal analgesia, continuous postoperative analgesia with catheter insertion and use of perineural additives have been advocated to minimize rebound pain [31, 34]. Since this survey did not capture the surgeons’ prescription, postoperative analgesia the patient received or the pain score, it is impossible to gauge the adequacy of pain relief.

The outcome metrics performed well, with the exception of cumulative dose of LA exceeding toxic limit in the instances where a mixture of LA was administered. The conversion rate to GA was 8.36%. Even though this appears higher than that reported in a multicentric study (conversion rate of 0.1%), it is important to note that 19% (321/1674) of their participants also received LA in the surgical field [35]. Although unexplored in this study, contributory factors for conversion to GA may include abnormal sonoanatomy, obesity, drug efficacy and expertise of the anesthetist.

In our study, toxic limit was not exceeded when a single agent was used. However, with a mixture, the cumulative dose of LA exceeded the toxic limit in 112/306 (36.6%) participants. Gadsden et al., demonstrated that combining mepivacaine and bupivacaine did not result in faster onset of block [36], thus disproving a common myth. Mixing LA results in an additive dose which is likely to exceed toxic limits [37, 38].

While a number of domains (Additional File, Table A) assessed were within the acceptable benchmark (Fig: 1), it is clear that individual components related to structure, process and outcome of PNB have potential for improvement. Most of the previous studies have been national surveys on regional anesthesia, rather than exclusively on PNB [39–42]. A national survey from an upper middle-income country nearly a decade ago reported landmark guided technique of PNB, a lack of training and lack of intralipid in majority of cases; however, this may have changed since then [39]. A recent survey from an LMIC highlighted deficiencies in monitoring and need for regional anesthesia training programs [40]. In contrast, a survey from a high-income country reported standard monitoring was used regularly; however, a deficiency noted was the non-uniform use of sterile USG cover [41]. Another survey from a high-income country highlighted the absence of block rooms as a major limitation along with inadequate training [42]. It is apparent from these that the deficiencies vary widely across economic regions. While surveys are excellent at highlighting overarching lacunae, they are prone to reporter bias and cannot comprehensively analyze the standards of practice. The strength of the current study is that it highlights the need to monitor quality and safety of PNB, so as to ensure continued improvement– one cannot improve what one does not measure. To this end, Table 4 summarizes strategies to improve individual components, and thereby improve overall safety and quality of PNB.

**Table 4:** Strategies to improve safety, quality, and efficacy of peripheral nerve blocks

Deficiency identified	Recommendation for improvement
Pre-block preparation - Resuscitation drugs and equipment availability Standard monitors Intralipid	Periodic updates and continuing medical education for anesthetists [19, 20] Mandatory pre-block checklist [21] Visual memory aids in block area [22] Audits as quality checks [26] Knowledge and attitude of anesthetists to understand reasons for current practice and effect change.
Safety concerns with PNB administration in ill-equipped area	Collaboration with stakeholders for infrastructure and resources Establishment of dedicated “block room” [16]
SB4YB procedural variation	Updates on correct execution of SB4YB Visual memory aids in block vicinity [22] Empower operating theatre assistants to participate in and execute the procedure. Audits as quality check [26]
Aseptic precautions	Reinforce aseptic precautions through updates. Explore factors influencing non-compliance, understand perceived barriers and study behavioral influences. Collaboration with administration to ensure availability of appropriate resources. Visual memory aids in block areas [22] Pre-block checklist [21] In-depth analysis and audit on individual components such as hand washing and skin preparation
Safety concerns regarding PNI.	Collaboration with administration to ensure availability of injection pressure monitor. Consider cost-effect alternative such CAIT for injection pressure monitoring [29] Encourage combined use of PNS and USG through updates [2, 14]
Exceeding toxic limit of LA	Encourage use single local anesthetics [36] Reduce maximum dose to half if mixing drugs [37, 38]
Postoperative analgesia	Standardize postoperative analgesia by either surgeon or anesthetist, not both. Acute pain care teams to assess adequacy of pain relief. Institute multimodal analgesia [31, 34] Consider use of perineurial additives [31, 34] Consider continuous block techniques with catheter [31, 34] Quality improvement initiatives to optimize delivery of pain relief.

Abbreviations: PNB- peripheral nerve blocks, SB4YB- Stop Before You Block, PNI- Peripheral nerve injury, CAIT- Compressed Air Injection Technique, PNS- Peripheral nerve stimulator, USG- ultrasound, LA- local anesthetics

The study is limited in the singularity of the center in which it was carried out. Additionally, the level of training in PNB for the anesthetists administering the PNB was not assessed, neither were the barriers to implementation of best practice. Further studies to understand knowledge and attitude of anesthetists and stakeholders are required to identify barriers to implementation of best practices. Frequent academic updates, formulating institutional policies with periodic quality checks, upgradation of infrastructure (where feasible) and collaboration with stakeholders for improving SoP will greatly enhance safety, quality, and efficacy of PNB administration in the low-resource setting. Quality improvement initiatives using the plan-do-study-act model could be initiated to improve the SoP for PNB.

## Conclusion

Standards of practice with regard to the structure, process and outcome were partly achieved for PNB at this tertiary care center in a low-middle income country. Parameters related to presence of venous access, use of PPE, use of ultrasound, use of short bevel needle, rate of conversion to GA and immediate post-block complications exceeded the benchmark, while areas with average quality were related to availability of resuscitation equipment, following “stop before you block”, site preparation aseptic procedures, use of additives in block and maintaining drug dose within cumulative toxic doses. Areas that required improvement were related to the availability of resuscitation drugs and postoperative multimodal analgesia prescription. Strategies that could improve SoP have been proposed and will need to be evaluated in future quality improvement initiatives.

## Electronic supplementary material

Below is the link to the electronic supplementary material.

Supplementary Material 1

## Data Availability

The datasets used and/or analyzed during the current study are available from the corresponding author on reasonable request.

## Abbreviations

LMIC: Low-Middle Income Countries
SoP: Standard of Practice
PNB: Peripheral Nerve Block
USG: Ultrasound
SD: Standard Deviation
PHB: Preoperative Holding Bay
GA: General Anesthesia
LAST: Local Anesthetic Systemic Toxicity
BMI: Body Mass Index
ASA: American Society of Anesthesiologists
OR: Odds Ratio
CI: Confidence Interval
WSB: Wrong-Sided Blocks
PNS: Peripheral Nerve Stimulator
PNI: Peripheral Nerve Injury
CAIT: Compressed Air Injection Technique
LA: Local Anesthetic
