# Supplementary material for: Standards of practice for peripheral nerve blocks at a tertiary care center in a low-middle income country– a prospective observational study
Source: BMC Anesthesiol. 2025 May 24;25:261. doi: 10.1186/s12871-025-03125-8 (PMC12102975; doi:10.1186/s12871-025-03125-8)
Supplement: Supplementary file 1 — Supplementary Material 1 [file 12871_2025_3125_MOESM1_ESM.docx]

**Additional File**

**TABLE A: Domains of data collection mapped to Donabedian model.**

| **DOMAIN** | **MAPPING TO DONABEDIAN MODEL** |
| --- | --- |
| **Domain 1: Demographic data**   - Age - Gender - Height and weight | **-**  **-**  **-** |
| **Domain 2: Preoperative assessment**   - American Society of Anesthesiologists - Physical Status (ASA PS) - Co-morbid illnesses - Name of surgery - Nature of surgery - elective/ emergency - Plan of anesthesia - Preoperative laboratory values: platelet count/ Prothrombine Time(PT) with INR/ activated Partial Prothrombin Time(aPTT) - Any antithrombotic medications? If yes, details and whether discontinued. | **-**  **-**  **-**  **-**  **-**  **-**  **-** |
| **Domain 3: Preparation for the block**   - Anesthesia machine checked and in working order - Oxygen source - Oxygen tubing - Manual resuscitator / self-inflating bag - Face mask - Intubation tray with working laryngoscopes - Resuscitation drugs: Atropine/mephentermine/ glycopyrrolate/ adrenaline - Midazolam - Intralipid yes/no - IV access for patient - Monitors attached Non Invasive Blood Pressure (NIBP)/pulse oximeter/ Electrocardiogram (ECG) leads | **Structure**  **Structure**  **Structure**  **Structure**  **Structure**  **Structure**  **Structure**  **Structure**  **Structure**  **Process**  **Process** |
| **Domain 4: Aseptic precautions**   - Cap - Mask - Gloves: sterile/ non-sterile - Sterile gown - Skin preparation: spirit/ betadine/ chlorhexidine/ butadiene followed by spirit - Sterile drapes for field - Sterile cover for ultrasound probe - Lubricant gel: sterile/ non-sterile | **Process**  **Process**  **Process**  **Process**  **Process**  **Process**  **Process**  **Process**  **Process** |
| **Domain 5: Details of block administration**   - Directly observed? Yes/ No - Location of block administration: Inside operating theatre/ preoperative holding bay*/others^+^ - Block(s) administered. - “STOP BEFORE YOU BLOCK” performed yes/no - Technnique used: Landmark/ nerve stimulator/ Ultrasonography (USG) / USG+ nerve stimulator - If USG - in-plane or out-of-plane? - Type of needle used: Short bevel block needle/ IV canal/ spinal needle/ hypodermic needle/ others - Catheter inserted? Yes/No - Drug(s) used: Drug/ Volume/ Concentration - Additives used? Yes/ No. If yes, drug and dose - Condition of block administration: Awake, non-sedated / Under sedation/ Under GA - If sedation used, drug and dose - If planned under block alone, any conversion to GA? - If conversion to GA, reason? Failed block/ patchy block/ change of surgical plan by surgeon requiring additional analgesia not covered by block/ block faded with the course of surgery/ patient request/ uncooperative patient / others - Any immediate post block administration/ intraoperative complications: yes/no | **-**  **-**  **-**  **Process**  **Process**  **-**  **Process**  **-**  **-**  **Process**  **-**  **-**  **Outcome**  **-**  **Outcome** |
| **F. Domain 6: Post operative analgesia prescription**   - Postoperative analgesia prescription present? Yes/ No - If Yes, drug, dose, and frequency | **Process**  **Process** |

*Preoperative holding bay: Holding area for patients before the surgery. Wall mounted oxygen source was available; however, availability of monitoring equipment and drugs was variable. ^+^Others: Locations outside operating theatre other than preoperative holding bay.

**Abbreviations:** PT - prothrombin time, aPTT- activated partial thromboplastin time, NIBP- non-invasive blood pressure, ECG- electrocardiogram, USG- ultrasound, IV- intravenous, GA- general anesthesia

**Table B: Details of anatomical site of block administration**

| **Site** | **n, (%)** |
| --- | --- |
| Upper limb | 289 (74.8%) |
| Lower limb | 79 (20.4%) |
| Upper limb and lower limb | 1 (0.2%) |
| Breast | 9 (2.3%) |
| Abdomen | 4 (1.03%) |
| Clavicle | 3 (0.7%) |
| Bladder | 1 (0.25%) |
